# Systematic relevance of pollen morphology in tribe Hylocereeae (Cactaceae)

**DOI:** 10.3897/phytokeys.128.35842

**Published:** 2019-08-19

**Authors:** Catalina Ruiz-Domínguez, Andrew P. Vovides, Victoria Sosa

**Affiliations:** 1 Biología Evolutiva, Instituto de Ecología, A.C. Carretera Antigua a Coatepec 351, 91073 El Haya, Xalapa, Veracruz, Mexico Instituto de Ecología Xalapa Mexico

**Keywords:** *
Hylocereus
*, pantocolpate, *
Selenicereus
*, stenopalynous, tricolpate

## Abstract

Hylocereeae is one of the nine tribes in the subfamily Cactoideae (Cactaceae), for which the limits and recognition of genera have been controversial. Essentially, this group comprises epiphytic and hemiepiphytic genera with stems modified as climbing structures. The aim of this paper is to examine pollen attributes in representative species of genera of Hylocereeae, focusing on *Selenicereus* whose current circumscription comprises *Hylocereus* and three *Weberocereus* species, to find whether significant potentially apomorphic and/or autapomorphic systematic characters can be discovered. Utilizing SEM and light microscopy, 25 pollen characters were observed and measured. Tribe Hylocereeae is stenopalynous, with pollen grains isopolar and radially symmetrical monads, mostly tricolpate, except in *Kimnachia*, *Pseudoripsalis* and *Weberocereus*, whose pollen grains are pantocolpate. Seven attributes (five qualitative and two continuous) exhibited useful variation and were coded. The character of brevicolpate pollen grains was shared by *Kimnachia ramulosa* and *Pseudorhipsalis amazonica*. Convex quadrangular outline in the polar view was shared by *Weberocereus tunilla* and *S. glaber*. The absence of spinules on the exine was shared by *S. minutiflorus* and *S. stenopterus.* The largest pollen grain, found in *Selenicereus megalanthus*, might be correlated with polyploidy. *Selenicereus* is the taxon with the highest variation in pollen attributes, including species with an exine with or without spinules and variable polar area index and shape (subprolate or oblate-spheroidal).

## Introduction

**Figure 1.**
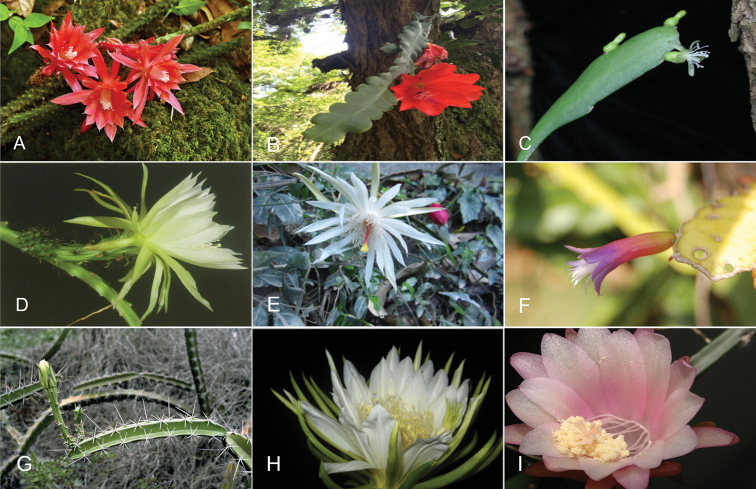
Morphological variation in tribe Hylocereeae. **A***Aporocactus martianus* (Photo S. Avendaño) **B***Disocacus ackermannii* (Photo C. Ruiz) **C**Kimnachia ramulosa subsp. ramulosa (From Flora de Nicaragua, O.M. Montiel) **D***Selenicereus atropilosus* (From Hunt 2006) **E***Epiphyllum hookeri* (Photo C. Ruiz) **F***Pseudorhipsalis amazonica* (From Mobot - Hammel 24524) **G***Acanthocereus tetragonus* (Photo C. Gómez-Hinostrosa) **H**Selenicereus (Hylocereus) undatus (Photo C. Ruiz) **I***Weberocereus tunilla* (From Mobot – B. Hammel 22442).

Hylocereeae is one of the nine tribes in subfamily Cactoideae (Cactaceae), in which the limits and recognition of genera have been controversial (Britton and Rose 1920; Buxbaum 1958; Barthlott and Hunt 1993; Bauer 2003; Korotkova et al 2017). With the exception of *Acanthocereus*, this group comprises epiphytic and hemiepiphytic genera with approximately 82 species with stems modified as climbing structures (Barthlott and Hunt 1993). It includes ornamentals in genera such as *Selenicereus*, *Epiphyllum* and *Disocactus*, whose flowers are night-blooming and known as queen of the night. It also includes *Hylocereus* with several species producing edible fruits known as dragon fruit, pitaya or pitahaya, which are cultivated in tropical areas around the world (Anderson 2001). Species in the tribe share characters such as angled stems and branches, with ribs, furrows or rarely smooth, aerial roots and usually spiny or hairy ovary areoles (Fig. 1) (Bauer 2003; Hunt et al. 2006).

Five taxonomical studies have introduced classifications for tribe Hylocereeae in the last century. First, the group was considered a subtribe in tribe Cereeae by Britton and Rose (1920) recognizing 9 genera with 48 species (*Aporocactus*, *Deamia*, *Hylocereus*, *Mediocactus*, *Selenicereus*, *Strophocactus*, *Weberocereus*, *Werckleocereus* and *Wilmattea*). Later, Buxbaum (1958) raised this group to the tribe level, tribe Hylocereeae, adding three genera to the previous classification: *Disocactus*, *Epiphyllum* and *Pseudorhipsalis*; however, *Deamia* was not accepted. The subsequent classification by Barthlott and Hunt (1993) differed notably from the preceding taxonomies, with the tribe comprising only six genera: *Discocactus*, *Epiphyllum*, *Hylocereus*, *Pseudorhipsalis*, *Selenicereus* and *Weberocereus*. The most recent classification based on the monophyletic groups identified by a plastid molecular phylogeny, recognized eight genera in tribe Hylocereeae: *Acanthocereus* (including Peniocereus subg. Pseudoacanthocereus), *Aporocactus*, *Disocactus*, *Epiphyllum*, *Kimnachia* (a new genus for *Pseudorhipsalis ramulosa*), *Pseudorhipsalis*, *Selenicereus* (incorporating *Hylocereus*, *Weberocereus alliodorus*, *W. glaber* and *W. tonduzii*) and *Weberocereus*. In comparison with previous definitions of tribe Hylocereeae, *Deamia* and *Strophocactus* were excluded, and *Acanthocereus* was added (Korotkova et al. 2017).

The aim of this paper is to examine pollen attributes in representative species of genera of tribe Hylocereeae focusing on the current concept of *Selenicereus* that includes *Hylocereus* and three species of *Weberocereus* to find whether potentially apomorphic and/or autapomorphic character states can be discovered. Traditionally, pollen has provided valuable and significant characters in plant taxonomy (Larson and Skvarla 1962; Nowicke and Skvarla 1979; Ferguson 1985), and in particular for Cactaceae, pollen characters continue to be useful taxonomically (e.g. Anderson and Skillman 1984; Rose and Barthlott 1994; Halbritter et al. 1997; Aguilar-García et al. 2012; Gonzaga et al. 2019; Mouga et al. 2019). Furthermore, pollen attributes have been used as a tool to clarify the taxonomy of diverse and complex angiosperm groups such as Poaceae (Dórea et al. 2017), and of difficult genera such as *Rosa* or *Psidium* (Wrońska-Pilarek and Jagodziński 2011; Tuler et al. 2017). Likewise, pollen morphology has been useful in systematic determinations at generic level in large families such as Asteraceae (Zhao et al. 2000), Liliaceae (Du et al. 2014), Ericaceae (Wrońska-Pilarek et al. 2018), and Bignoniaceae (Burelo-Ramos et al. 2009).

Leuenberger (1976) compiled the most complete description of pollen morphology of 600 cactus species, and found that aperture ratio was one of the most variable and useful characters at different taxonomic levels, from subfamily to genus. In addition, Kurtz (1963) in his study of pollen in Cactaceae – which included several genera in Hylocereeae – identified relevant variation in pollen size in *Hylocereus* and *Selenicereus*, differences in the number of furrows in a number of genera such as *Weberocereus*, and useful variation in pollen sculpture in the length of spinules and perforation of the exine in several genera. Likewise, the identification of species in Cactaceae using pollen in countries such as Brazil, Peru and Argentina found useful characters such as variation in size, shape, and exine thickness to determine taxa at tribe and genus levels (Santos and Watanabe 1996; Santos et al. 1997; Garralla and Cuadrado 2007; Cuadrado and Garralla 2009; Lattar and Cuadrado 2010; De la Cruz et al. 2013; Miesen et al. 2015; Cancelli et al. 2017).

The study of the pollen of tribe Hylocereeae is part of our current project on the evolution and systematics of the *Hylocereus* clade. We aim to incorporate pollen characters with morphological and molecular data to better understand the limits and phylogenetic position of this group, along with phylogenetic relationships of its species, the evolution of chemical and fruit characters. In this paper, the pollen attributes of 27 representative taxa of the genera comprising tribe Hylocereeae, with a main focus in the current concept of *Selenicereus* (including *Hylocereus* and three *Weberocereus* species), are examined to determine whether character states are shared or are exclusive in these taxa.

## Materials and methods

### Sampling

Pollen grains of 27 species of tribe Hylocereeae, corresponding to 8 genera according to classifications of this group, were collected (Korotkova et al. 2017). Anthers were either collected directly in the field and preserved in envelopes or from herbarium specimens. Representative species in the following genera were sampled (No. spp. sampled/No. spp. in the genus, *sensu*Korotkova et al. 2017): *Acanthocereus* (2/13); *Aporocactus* (1/2); *Disocactus* (2/14); *Epiphyllum* (2/10); *Kimnachia* (1/1); *Pseudorhipsalis* (1/5); *Weberocereus* (1/6). *Selenicereus* (comprising *Hylocereus* and *Weberocereus pro parte*) (17/31). The species vouchers are included in the descriptions of pollen morphology

### Pollen preparation

The acetolysis method proposed by Erdtman (1960) was used for processing the pollen grains for observation. For difficult material such as collapsed grains or delicate pollen, the suggestions of Fonnegra (1989) were implemented. Pollen grains were mounted in jelly and sealed. For observing pollen with scanning electron microscope (SEM), the material was dried at critical point and sputter coated in palladium gold (Boyde and Wood 1969). SEM observations were made and electromicrographs taken with a Jeol JSM-5600LV scanning electron microscope.

### Qualitative pollen characters

**Figure 2.**
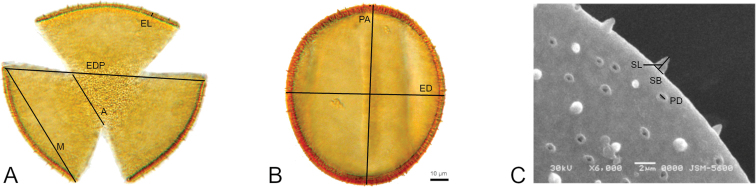
Quantitative and qualitative characters from pollen grains: **A, B** optical Microscope Photographs (800×) **A** polar view: Apocolpium (A), Mesocolpium (M), Equatorial diameter in polar view (EDP), Exine length (EL), Amb, Polarity, Symmetry, Aperture length, Polar Area Index (PAI) = A/EDP **B** equatorial view: Equatorial diameter (ED), Polar axis (PA), Pollen unit, Aperture (number, position, character), Pollen type, Shape class (PE) = PA/ED **C** scanning Electron Microscope Photograph (6000x); Exine Ornamentation, Spinule length (SL), Spinule base (SB), Perforation diameter (PD).

For the species studied, fourteen qualitative characters were coded: 1) shape of pollen grain, 2) type of polar area, 3) aperture (based in polar area index = apocolpium/ equatorial diameter in polar view), 4) outline of the pollen grain polar view (amb), 5) pollen unit, 6) pollen type (according to polar axis longitude), 7) polarity, 8) aperture class (colpate or brevicolpate), 9) number of colpi, 10) symmetry, 11) tectum (perforate or imperforate), 12) exine (tectate or semitectate), 13) exine spinules (present or absent), 14) margo (present or absent) (Fig. 2). The terminology for pollen grain characters follows Punt et al. (2007), and character denomination follows Erdtman (1952) and Salgado-Labouriau (1966). The evaluation of attributes in pollen grains was based on one specimen, following Wrońska-Pilarek et al. (2014). Their study corroborated that the number of pollen grains measured is more important than the number of analyzed specimens, indicating that a sample should contain at least 25 pollen grains (Wrońska-Pilarek et al. 2014).

### Morphometric pollen characters

Eleven morphological continuous pollen characters of the studied species were measured, including 1) equatorial diameter in polar view, 2) apocolpium (area delimited by lines connecting the apices of the colpi at the pole of the pollen grain), 3) mesocolpium (area delimited by lines between the apices of adjacent colpi), 4) polar axis in equatorial view, and 5) equatorial diameter in equatorial view. They were measured for a maximum of 25 pollen grains from at least three preparations of a single specimen for each species, with 800× optical zoom under a Carl Zeiss Fomi III Optical Microscope, equipped with a Cannon Power Shot G9 digital camera. Additionally, under 1250× optical zoom, 10 pollen grains were observed to measure 6) exine thickness (Fig. 2). For further analysis, SEM electromicrographs on 10 pollen grains for each species with 6000× magnification SEM analysis was performed on acetolyzed and non-acetolyzed pollen material. With SEM, the following exine characters were measured: 7) spinule length, 8) spinule base, and 9) perforation diameter. In addition, the following ratios were estimated: 10) PAI (Polar Area Index) PAI= Apocolpium/ Equatorial diameter in polar view and 11) PE (Shape class) PE=Polar axis/Equatorial diameter in equatorial view. Character measurements from optical microscopy were obtained with the software Axio Vision ver. 4.7.2, and characters from SEM were acquired using ImageJ 1.45 software (Abramoff et al. 2004) (Fig. 2). To identify character states, every continuous character was coded following Almeida and Bisby (1984), ordered in boxplot diagrams by median values, and examined for dips or gaps. Gaps based on the first and third quartiles are codified as discontinuities and the corresponding character states are assigned.

### Exploratory multivariate analyses

A principal component analysis (PCA) was performed using the packages Factoextra and FactoMine in R (R Development Core Team 2018) to reduce the dimensionality of phenotypic variation and summarize the variables that are correlated. PCA was carried out to identify the characters that explained the greatest proportion of the variability and to identify pollen grains occupying different spaces.

## Results

The taxa studied in tribe Hylocereeae are stenopalynous, i.e. there is slight variation in pollen grains. They are isopolar and radially symmetrical monads, mostly tricolpate, with the exception of *Kimnachia*, *Pseudoripsalis* and *Weberocereus*, whose pollen grains are pantocolpate, with 12 to 15 colpi.

In the following paragraphs detailed descriptions of the pollen grains are provided.

### Acanthocereus

Taxon classificationPlantaeCaryophyllalesCactaceae

(Engelm. ex A. Berger) Britton & Rose

1da777c3-93c8-5b8a-92b0-50d323bb6446

#### Pollen.

**Figure 3.**
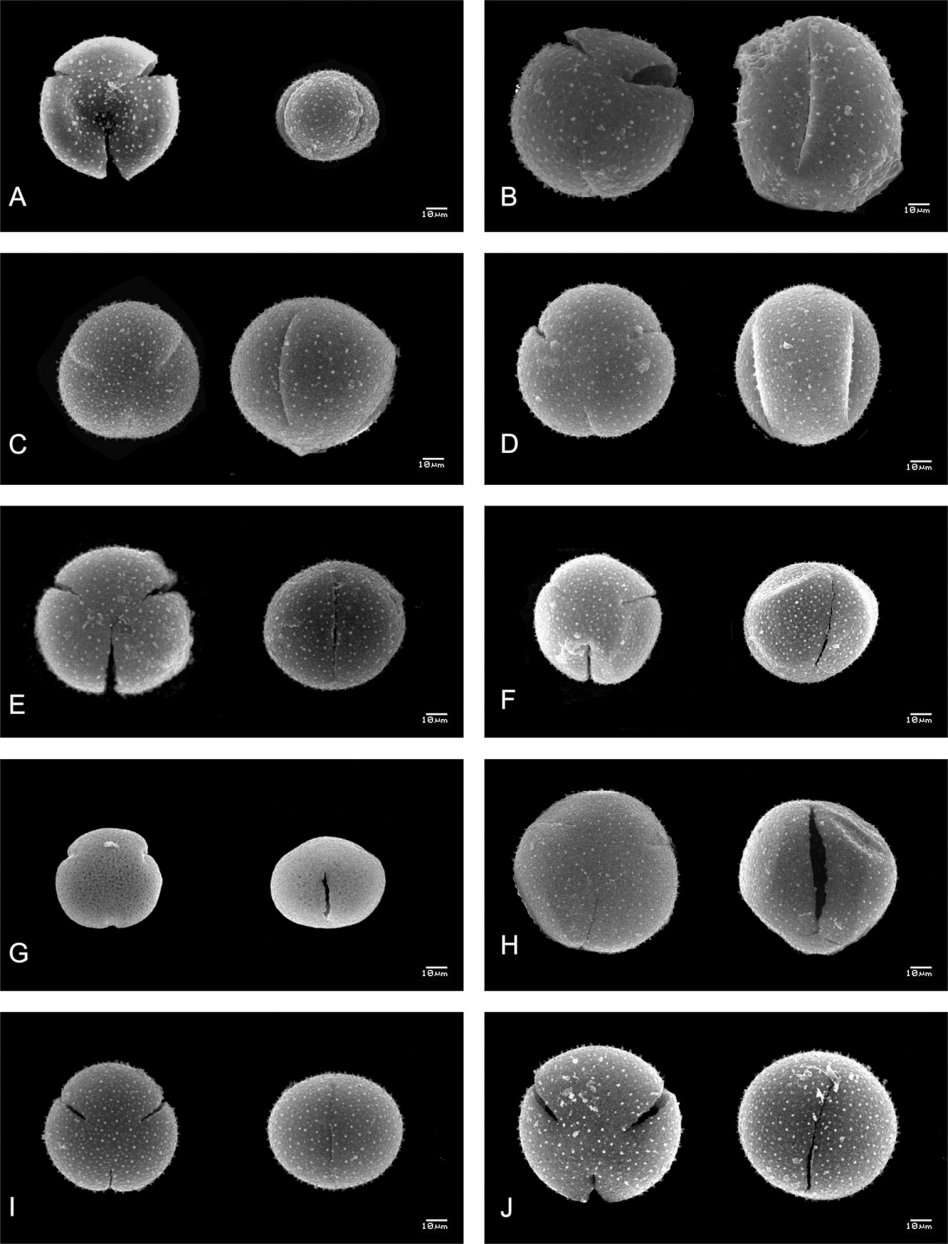
Pollen grains of tribe Hylocereeae (Scanning Electron Microscope photographs). Left polar view, right equatorial view **A***Acanthocereus tetragonus* (C. Ruiz et al. 576) **B***Disocactus ackermanii* (R. Torres et al. 309) **C***Epiphyllum oxypetalum* (C. Ruiz et al. 640) **D***Selenicereus costaricensis* (C. Ruiz et al. 555) **E***S. escuintlensis* (C. Ruiz et al. 635) **F***S. guatemalensis* (M. Véliz et al. 20227) **G***S. minutiflorus* (C. Ruiz et al. 627) **H***S. ocamponis* (M. Cházaro 7334) **I***S. polyrhizus* (C. Ruiz et al. 566) **J***S.* sp. (C. Ruiz et al. 608).

trizonocolpate, radially symmetrical, isopolar with circular contour in polar view. **Shape**: varies from subprolate to oblate-spheroidal (P/E=0.97–1.29). **Apertures**: 3, colpate, large; polar area of medium size (PAI=0.31–0.40). **Measurements**: pollen grains of large size (49.02)56.81(68.53) × (49.89)63.62(79.4) μm in equatorial view; exine thickness (2.41)3.29(4.19) μm. **Ornamentation**: smooth surface with tectum perforate, ornated with spinules of (1.05)1.43(1.76) μm length × (1.08)1.37(1.83) μm diameter in base; perforations (0.17)0.25(0.33) μm in diameter (Figure 3A).

#### Species examined.

*Acanthocereus tetragonus* (L.) Hummelinck (Colombia, Valle del Cauca. C. Ruiz et al. 576 CUVC); *Acanthocereus chiapensis* Bravo (México, Chiapas. C. Gómez-Hinostrosa et al. 2325 MEXU).

### Aporocactus

Taxon classificationPlantaeCaryophyllalesCactaceae

Lemaire

f74b4d4d-17dc-5a96-9cd3-5006a90cc4cd

#### Pollen.

trizonocolpate, radially symmetrical, isopolar with circular contour in polar view. **Shape**: subprolate (P/E=1.15). **Apertures**: 3, colpate, large; polar area of medium size (PAI=0.36). **Measurements**: pollen grains large to very large, (75.98)93.62(110.47) × (80.52)106.91(117.69) μm in equatorial view; exine thickness (3.16)3.66(4.34) μm. **Ornamentation**: smooth surface with tectum perforate, ornated with spinules of (1.31)1.64(1.93) μm length × (1.18)1.53(2.10) μm diameter in base; perforations (0.14) 0.23(0.34) μm in diameter.

#### Species examined.

*Aporocactus martianus* (Zucc.) Britton & Rose. (México, Veracruz. H. Narave et al. 308 XAL).

### Disocactus

Taxon classificationPlantaeCaryophyllalesCactaceae

Lindley

cc230b89-f890-5a2f-932c-9c9f5481ca8f

#### Pollen.

trizonocolpate, radially symmetrical, isopolar with circular contour in polar view. **Shape**: varies from subprolate to prolate-spheroidal (P/E=1.13–1.18). **Apertures**: 3, colpate, large; polar area of medium size (PAI=0.33–0.34). **Measurements**: pollen grains large to very large, (80.36)97.51(116.99) × (99.25)112.18(125.04) μm in equatorial view; exine thickness (2.24)3.27(3.99) μm. **Ornamentation**: smooth surface with tectum perforate, ornated with spinules of (1.22)1.63(2.50) μm length × (1.07)1.45(2.06) μm diameter in base; perforations (0.30)0.61(0.98) μm in diameter (Figure 3B).

#### Species examined.

*Disocactus ackermanii* (Haw.) Ralf Bauer (México, Oaxaca. R. Torres et al. 309 MEXU); *Disocactus speciosus* (Cav.) Barthlott (México, Edo de México. J. Canek Ledesma 2211 MEXU).

### Epiphyllum

Taxon classificationPlantaeCaryophyllalesCactaceae

Haworth

f7de5e4c-d2b3-52fa-96fc-a77983e137c7

#### Pollen.

trizonocolpate, radially symmetrical, isopolar with circular contour in polar view. **Shape**: varies from oblate-spheroidal to prolate-spheroidal (P/E=0.90–1.06). **Apertures**: 3, colpate, large; polar area of medium size (PAI=0.32–0.46). **Measurements**: pollen grains large, (69.33)88.76(111.93) × (74.43)86.49(105.34) μm in equatorial view; exine thickness (2.24)2.73(3.67) μm. **Ornamentation**: smooth surface with tectum perforate, ornated with spinules of (1.22)1.69(2.02) μm length × (0.97)1.26(1.57) μm diameter in base; perforations (0.32)0.51(0.65) μm in diameter (Figure 3C).

#### Species examined.

*Epiphyllum oxypetalum* (DC.) Haw. (Guatemala, Sacatepéquez. C. Ruiz et al. 640 BIGU); *Epiphyllum thomasianum* (K. Schum.) Britton & Rose (Guatemala, Sacatepéquez. C.K. Horich 572922 MEXU).

### Kimnachia

Taxon classificationPlantaeCaryophyllalesCactaceae

S. Arias & N. Korotkova

efba07e0-92da-5712-b61c-c2037cc1495c

#### Pollen.

**Figure 4.**
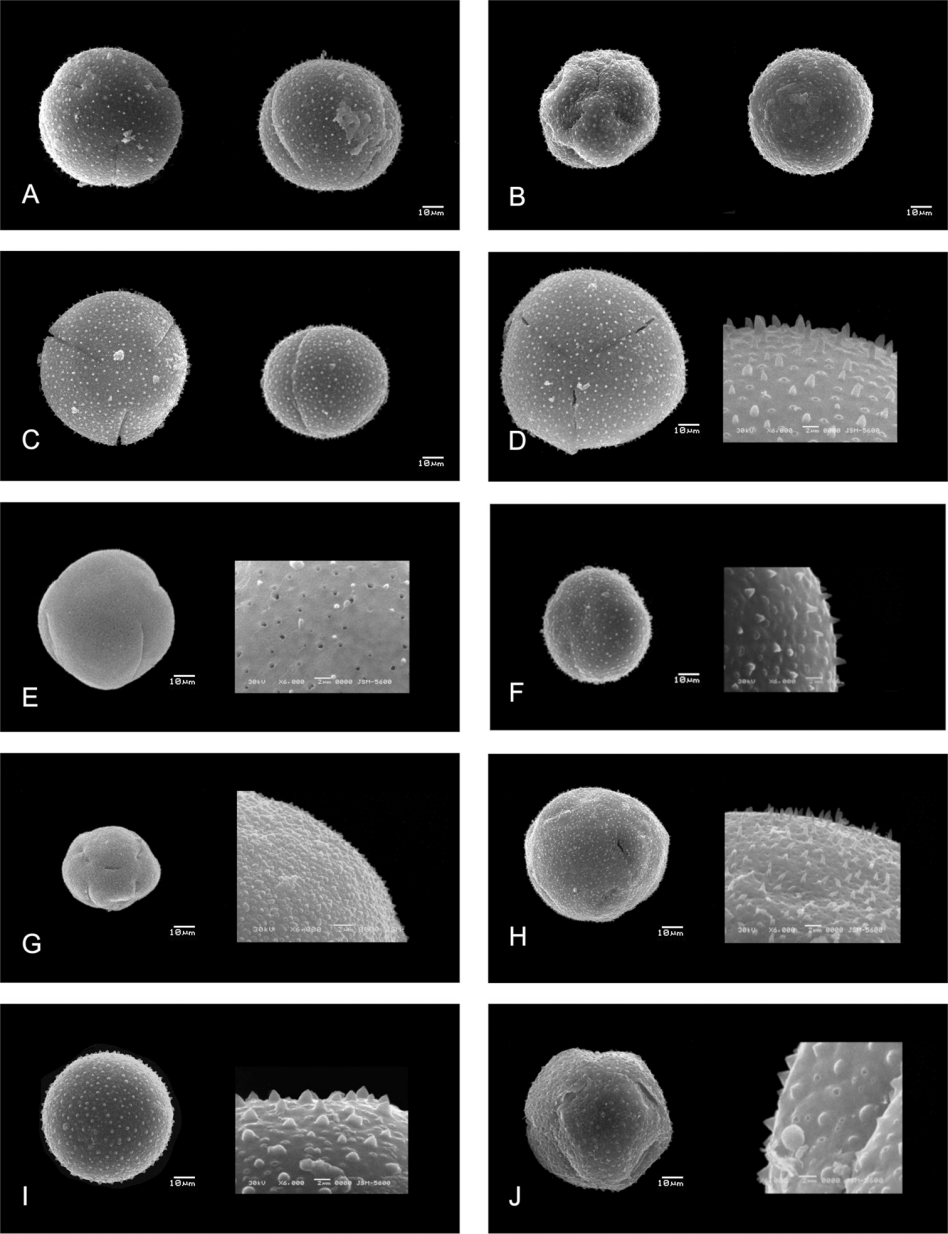
Pollen grains of tribe Hylocereeae (Scanning Electron Microscope photographs). **A–D**: Left polar view, right equatorial view **A***S. undatus* (C. Ruiz et al. 560) **B***Weberocereus tunilla* (C.K. Horich BGA 58344) **C***S. setaceus* (H. Bravo et al. 2755). **D–J**: left polar view, left details of spinules and perforations **D***S. megalanthus* (C. Ruiz et al. 563) **E***S. stenopterus* (C.K. Horich s.n.) **F***S. triangularis* (C. Gómez-Hinostrosa 2110) **G***Kimnachia ramulosa* (L. Velásquez et al. 4884) **H***Pseudorhipsalis amazonica* (E. Gudiño 145) **I***S. alliodorus* (A. Ruiz Velazco et al. 86) **J***S. glaber* (C.K. Horich BGA 57239).

pantocolpate, radially symmetrical, isopolar with circular contour in polar view. **Shape**: prolate-spheroidal (P/E=1.07). **Apertures**: 15, brevicolpate, very large; polar area small (PAI=0.05). **Measurements**: pollen grains medium-sized to large, (43.95)49.14(55.73) × (48.04)52.45(58.32) μm in equatorial view; exine thickness (3.10)3.84(4.74) μm. **Ornamentation**: smooth surface with tectum perforate, ornated with spinules of (0.19)0.33(0.43) μm length × (0.34)0.45(0.55) μm diameter in base; perforations (0.09)0.18(0.35) μm in diameter (Figure 4G).

#### Species examined.

*Kimnachia ramulosa* (Salm-Dyck) S. Arias & N. Korotkova (Guatemala, Suchitepéquez. L. Velásquez et al. 4884 BIGU).

### Pseudorhipsalis

Taxon classificationPlantaeCaryophyllalesCactaceae

Britton & Rose

ab328040-eacf-5891-b771-3328d555f585

#### Pollen.

pantocolpate, radially symmetrical, isopolar with circular contour in polar view. **Shape**: oblate-spheroidal (P/E=0.99). **Apertures**: 12, brevicolpate, small; polar area large (PAI=0.62). **Measurements**: pollen grains large, (73.59)81.71(93.64) × (76.00)81.23(86.72) μm in equatorial view; exine thickness (2.85)3.19(3.48) μm. **Exine**: marginated. **Ornamentation**: smooth surface with tectum perforate, ornated with spinules of (0.64)0.78(0.99) μm length × (0.39)0.48(0.54) μm diameter in base; perforations (0.05)0.11(0.19) μm in diameter (Figure 4H).

#### Species examined.

*Pseudorhipsalis amazonica* (K. Schum.) Ralf Bauer (Ecuador, Napo. E. Gudiño 145 MEXU).

### Selenicereus

Taxon classificationPlantaeCaryophyllalesCactaceae

(A. Berger) Britton & Rose

67dd9c14-ced8-5b8c-a912-a8133189d874

#### Pollen.

trizonocolpate, radially symmetrical, isopolar with circular contour in polar view. **Shape**: prolate-spheroidal (P/E=1.06–1.07). **Apertures**: 3, colpate, large; polar area medium-sized (PAI=0.36–0.37). **Measurements**: pollen grains large to very large, (66.74)87.12(110.39) × (70.79)90.62(119.77) μm in equatorial view; exine thickness (2.19)2.68(3.39) μm. **Ornamentation**: smooth surface with tectum perforate, ornated with spinules of (1.09)1.58(2.07) μm length × (0.92)1.30(1.86) μm diameter in base; perforations (0.17)0.30(0.56) μm in diameter.

#### Species examined.

*Selenicereus grandiflorus* (L.) Britton & Rose (México, Veracruz. Rivera-Alarcón et al. 37 XAL); *Selenicereus hamatus* (Scheidw.) Britton & Rose (México, Veracruz. D. Jimeno-Sevilla 1079 XAL).

### Selenicereus (Hylocereus sect. Hylocereus

Taxon classificationPlantaeCaryophyllalesCactaceae

clade)

3ea20c0c-08dc-520a-b19a-17cc58bcd0f2

#### Pollen.

trizonocolpate, radially symmetrical, isopolar with circular contour in polar view. **Shape**: varies from suboblate to subprolate (P/E=0.86–1.18). **Apertures**: 3, colpate (brevicolpate in *S. minutiflorus*), large (small in *S. minutiflorus*); polar area medium-sized to large (PAI=0.30–0.59). **Measurements**: pollen grains of medium to very large size, (54.52)78.67(97.83) × (45.94)83.68(102.53) μm in equatorial view; exine thickness (1.66)2.95(4.10) μm. **Ornamentation**: smooth surface with tectum perforate, ornated with spinules (*S. minutiflorus* and *S. stenopterus* without spinules) of (1.06)1.47(2.13) μm length × (0.72)1.23(1.53) μm diameter in base; perforations (0.20)0.39(0.89) μm in diameter (Figures 3D, 3E, 3F, 3G, 3I, 3J, 4A, 4E, 4F).

#### Species examined.

*Selenicereus costaricensis* (F.A.C. Weber) Britton & Rose (Colombia, Valle del Cauca. C. Ruiz et al. 555 CUVC); *S. escuintlensis* Kimnach (Guatemala, Escuintla. C. Ruiz et al. 635 BIGU); *S. guatemalensis* (Eichlam ex Weing.) Britton & Rose (Guatemala, El Progreso. M. Véliz et al. 20227 BIGU); *S. minutiflorus* Britton & Rose (Guatemala, Izabal. C. Ruiz et al. 627 BIGU); *S. monacanthus* (Lemaire) Britton & Rose (Honduras, Francisco Morazán. C. Ruiz et al. 493 TEFH); *S. polyrhizus* (F.A.C. Weber) Britton & Rose (Colombia, Valle del Cauca. C. Ruiz et al. 566 CUVC); *Selenicereus* sp. (México, Oaxaca. C. Ruiz et al. 608 XAL); *S. stenopterus* (F.A.C. Weber) Britton & Rose (Costa Rica, Alajuela. C.K. Horich s.n. MEXU); *S. triangularis* (L.) Britton & Rose (México, Yucatán. C. Gómez-Hinostrosa 2110 MEXU); *S. undatus* (Haworth) Britton & Rose (Colombia, Valle del Cauca. C. Ruiz et al. 560 CUVC).

### Selenicereus

Taxon classificationPlantaeCaryophyllalesCactaceae

(Hylocereus: Salmdyckia clade)

9cebb032-150f-5420-98a8-fb5666a616e4

#### Pollen.

trizonocolpate, radially symmetrical, isopolar with circular contour in polar view. **Shape**: Prolate-spheroidal to subprolate (P/E=1.01–1.16). **Apertures**: 3, colpate, large; polar area medium-sized (PAI=0.30–0.40). **Measurements**: pollen grains large to very large, (81.09)89.11(127.4) × (83.13)98.23(129.26) μm in equatorial view; exine thickness (1.94)2.92(4.56) μm. **Ornamentation**: smooth surface with tectum perforate, ornated with spinules of (1.17)1.61(2.07) μm length × (0.81)1.20(1.53) μm diameter in base; perforations (0.23)0.46(0.70) μm in diameter (Figure 3H, 4C, 4D).

#### Species examined.

*S. megalanthus* (K. Schumann ex Vaupel) Ralf Bauer (Colombia, Valle del Cauca. C. Ruiz et al. 563 CUVC); *S. ocamponis* (Salm-Dyck) Britton & Rose (México, Michoacán. M. Cházaro 7334 MEXU); *S. setaceus* (Salm-Dyck ex DC) Ralf Bauer (Brazil, Rio de Janeiro. H. Bravo et al. 2755 MEXU).

### Selenicereus

Taxon classificationPlantaeCaryophyllalesCactaceae

(ex Weberocereus)

90d188a7-46c3-5066-9e7c-456a6d8fdfa8

#### Pollen.

trizonocolpate to pantocolpate, radially symmetrical, isopolar with circular or convex-quadrangular contour in polar view. **Shape**: prolate-spheroidal (P/E=1.04–1.12). **Apertures**: 3 or 12, brevicolpate to colpate; polar area medium-sized to large (PAI=0.41–0.60). **Measurements**: pollen grains large to very large, (50.9)77.50(100.2) × (66.79)82.21(100.86) μm in equatorial view; exine thickness (2.29)2.96(3.77) μm. **Ornamentation**: smooth surface with tectum perforate, ornated with spinules of (1.12)1.48(1.82) μm length × (1.13)1.44(1.91) μm diameter in base; perforations (0.17)0.29(0.50) μm in diameter (Figure 4I, 4J).

#### Species examined.

*Selenicereus alliodorus* (México, Oaxaca. Gómez-Hinostroza & H. M. Hernández) S. Arias & N. Korotkova (A. Ruiz Velazco et al. 86 MEXU); *S. glaber* (Eichlam) G.D. Rowley (Guatemala, Sacatepéquez. C.K. Horich BGA 57239 MEXU).

### Weberocereus

Taxon classificationPlantaeCaryophyllalesCactaceae

Britton & Rose

78dad3ab-269a-57c1-827a-9dd23cb1ab81

#### Pollen.

pantocolpate, radially symmetrical, isopolar with convex-cuadrangular contour in polar view. **Shape**: prolate-spheroidal (P/E=1.07). **Apertures**: 12–15, brevicolpate, small; polar area large (PAI=0.56). **Measurements**: pollen grains large, (77.07)82.94(89.61) × (82.67)88.73(98.94) μm in equatorial view; exine thickness (1.96)2.43(2.85) μm. **Ornamentation**: smooth surface with tectum perforate, ornated with spinules of (1.24)1.41(1.58) μm length × (1.28)1.65(1.91) μm diameter in base; perforations (0.17)0.28(0.45) μm in diameter (Figure 4B).

#### Species examined.

*Weberocereus tunilla* (F.A.C. Weber) Britton & Rose (Costa Rica, Cartago. C.K. Horich BGA 58344 MEXU).

#### Qualitatitative pollen characters.

Of the fourteen characters examined, five were identified as variable: Amb (the outline of a pollen grain seen in polar view), colpi number, aperture type (colpate or brevicolpate pollen), marginate exine (an area of the exine around an ectocolpous that is differentiated from the remainder of the exine by difference in thickness and the presence of spinules). The other qualitative characters were not variable (Table 1, Suppl. material 1: Table S1). Pollen grains of the studied species in tribe Hylocereeae are oblate-spheroidal, tricolpate with spinules of variable size (Figs 3 and 4). *Weberocereus*, *Pseudorhipsalis*, and *Kimnachia* differ from the rest of the tribe by having pollen grains with a small aperture, brevicolpate, with differences in the number of colpi as well. *Aporocactus*, *Acanthocereus*, *Disocactus*, *Epiphyllum*, and *Selenicereus* (including *Hylocereus*) have tricolpate pollen. In addition, two autoapomorphic characters were identified: convex-quadrangular outline in *Weberocereus*, marginate exine in *Pseudorhipsalis* (Figs 3, 4).

#### Continuous pollen characters.

**Figure 5.**
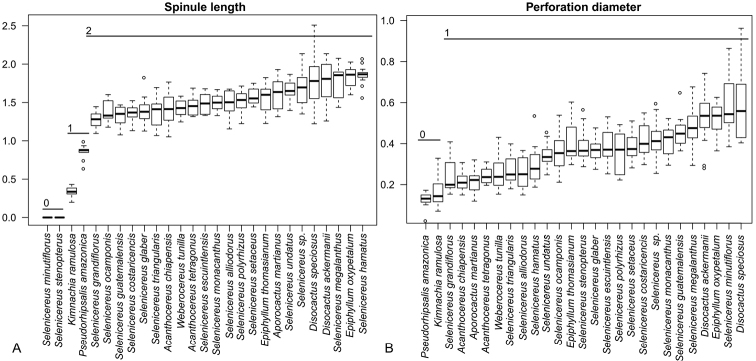
Box plots of two coded pollen characters, which are discriminant for genus in tribe Hylocereeae. **A** Spinule length **B** perforation diameter. Boxes represent the first and third percentiles and black lines indicate median values. Character states are indicated (0, 1 and 2) for each character and measurement range is indicated. Measurements are in µm.

The size of pollen grains for the 27 taxa examined varies from 55.47 to 154.42 μm in polar axis, and this is large according to Erdtman (1952). Boxplot diagrams ordered by median values found gaps for spinule length and perforation diameter based on the first and third quartiles (Fig. 5). These were coded based on the simple gap method by Almeida and Bisby (1984). Spinule length differentiates *Kimnachia* from *Pseudorhipsalis*. For *Selenicereus* (*Hylocereus* clade), spinule length was found to be polymorphic because it includes representatives lacking spinules and representatives with spinules of larger dimensions than those of *Kimnachia* and *Pseudorhipsalis* (Table 1).

**Table 1.** Qualitative and quantitative morphological characters of pollen for the representative species in the tribe Hylocereeae. Spinule length and perforation diameter are coded based on the simple gap method by Almeida and Bisby (1984). PE ratio =Polar axis/Equatorial Diameter; PAI ratio =Apocolpium/Equatorial diameter in polar view (Polar Area Index); Amb: Outline in polar view. Spinule length (0 = 0 µm, 1 = 0.33–0.78 µm, 2 = 1.27–1.86 µm); Perforation diameter (0 = 0.11–0.18 µm, 1 = 0.23–0.72 µm). P=present, A=Absent.

Species	PE ratio	Shape class	PAI ratio	PA type	Amb	Pollen type	Aperture	Margo	No of colpus	Spninules	Spinule length	Perforation diameter
*Acanthocereus chiapensis*	1.30	Subprolate	0.32	Medium	Circular	Large	Colpate	A	3	P	2	1
*Acanthocereus tetragonus*	0.97	Oblate-spheroidal	0.41	Medium	Circular	Large	Colpate	A	3	P	2	1
*Aporocactus martianus*	1.15	Subprolate	0.36	Medium	Circular	Very Large	Colpate	A	3	P	2	1
*Disocactus ackermanii*	1.19	Subprolate	0.34	Medium	Circular	Very Large	Colpate	A	3	P	2	1
*Disocactus speciosus*	1.13	Prolate-spheroidal	0.35	Medium	Circular	Very Large	Colpate	A	3	P	2	1
*Epiphyllum oxypetalum*	1.07	Prolate-spheroidal	0.32	Medium	Circular	Large	Colpate	A	3	P	2	1
*Epiphyllum thomasianum*	0.90	Oblate-spheroidal	0.47	Medium	Circular	Large	Colpate	A	3	P	2	1
*Kimnachia ramulosa*	1.07	Prolate-spheroidal	0.05	Small	Circular	Large	Colpate	A	15	P	1	0
*Pseudorhipsalis amazonica*	0.99	Oblate-spheroidal	0.63	Large	Circular	Large	Colpate	P	12	P	1	0
*Selenicereus alliodorus*	1.12	Prolate-spheroidal	0.41	Medium	Circular	Large	Colpate	A	3	P	2	1
*Selenicereus costaricencis*	1.18	Subprolate	0.38	Medium	Circular	Large	Colpate	A	3	P	2	1
*Selenicereus escuintlensis*	0.97	Oblate-spheroidal	0.37	Medium	Circular	Large	Colpate	A	3	P	2	1
*Selenicereus glaber*	1.05	Prolate-spheroidal	0.61	Large	Convex quadrangular	Large	Colpate	A	12	P	2	1
*Selenicereus grandiflorus*	1.07	Prolate-spheroidal	0.37	Medium	Circular	Very Large	Colpate	A	3	P	2	1
*Selenicereus guatemalensis*	1.04	Prolate-spheroidal	0.31	Medium	Circular	Large	Colpate	A	3	P	2	1
*Selenicereus hamatus*	1.06	Prolate-spheroidal	0.37	Medium	Circular	Large	Colpate	A	3	P	2	1
*Selenicereus megalanthus*	1.01	Prolate-spheroidal	0.40	Medium	Circular	Very Large	Colpate	A	3	P	2	1
*Selenicereus minutiflorus*	0.87	Suboblate	0.60	Large	Circular	Large	Colpate	A	3	A	0	1
*Selenicereus monacanthus*	1.11	Prolate-spheroidal	0.34	Medium	Circular	Large	Colpate	A	3	P	2	1
*Selenicereus ocamponis*	1.17	Subprolate	0.31	Medium	Circular	Very Large	Colpate	A	3	P	2	1
*Selenicereus polyrhizus*	1.07	Prolate-spheroidal	0.34	Medium	Circular	Large	Colpate	A	3	P	2	1
*Selenicereus setaceus*	1.10	Prolate-spheroidal	0.34	Medium	Circular	Very Large	Colpate	A	3	P	2	1
*Selenicereus* sp.	1.11	Prolate-spheroidal	0.35	Medium	Circular	Large	Colpate	A	3	P	2	1
*Selenicereus stenopterus*	1.17	Subprolate	0.38	Medium	Circular	Large	Colpate	A	3	A	0	1
*Selenicereus triangularis*	0.91	Oblate-spheroidal	0.45	Medium	Circular	Large	Colpate	A	3	P	2	1
*Selenicereus undatus*	1.04	Prolate-spheroidal	0.40	Medium	Circular	Large	Colpate	A	3	P	2	1
*Weberocereus tunilla*	1.07	Prolate-spheroidal	0.57	Large	Convex quadrangular	Large	Colpate	A	12, 15	P	2	1

#### Multivariate analyses.

**Figure 6.**
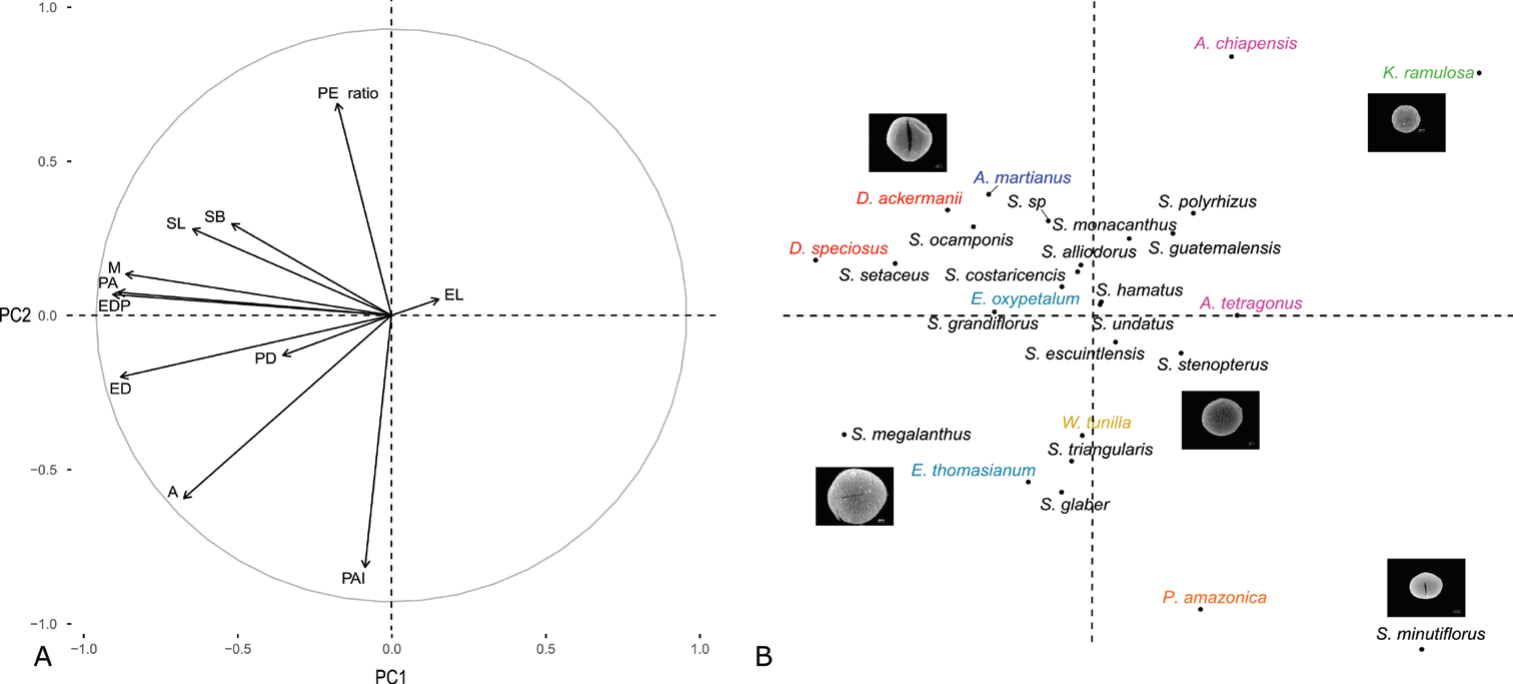
Principal Component Analysis graph. **A** Scatterplot displaying projections in a multidimensional space of the pollen grain characters: Apocolpium (A), Mesocolpium (M), Equatorial diameter in polar view (EDP) Exine length (EL), Polar Area Index (PAI), Equatorial diameter (ED), Polar axis (PA), Shape class (PE), Spinule length (SL), Spinule base (SB), Perforation diameter (PD). PC1 explains 44% of the variance and PC2 explains 18.4% of the variance **B** Sorting of the 27 species of tribe Hylocereeae, in relation to the morphometric variables of pollen grains.

The PCA graph displays projections of pollen characters in a multidimensional space in which the first two components explained 62.4% of the observed variance. PC 1 explains 44% of the variance and is associated with size (equatorial diameter, polar axis, and spinule dimensions), while PC 2, which explains 18.4% of the variance, is associated with proportions (PAI and PE ratios) (Fig. 6A). The length of the arrows in Figure 6A indicates adequate sampling for all characters, except for exine length (EL) and perforation diameter (PD). The size of pollen grains in the polar and in equatorial views had a positive correlation and, similarly, there is a negative correlation between the PE and PAI ratios, as expected in spherical forms. There is a negative correlation between exine length (EL) and the other measures. Figure 5B displays the species studied along the principal components in relation to pollen characters. The association of species is defined by the size of the pollen grain and its shape from prolate to oblate spheroidal). A core association of species is formed by representative species from all genera, including pollen mainly subprolate to prolate-spheroidal. The only species with no representatives in the core group and that appear as outliers are *Pseudorhipsalis amazonica* and *Kimnachia ramulosa*, with prolate to oblate pantocolpate pollen. Remarkably, Selenicereus (Hylocereus) megalanthus, along with *S. setaceus* are two of the species with prolate-spheroidal pollen grains with the largest polar axis; *S. minutiflorus K. ramulosa* have the smallest pollen grains (smallest polar axis). *Selenicereus minutiflorus* is the representative of Selenicereus (Hylocereus) with the unique characters of suboblate pollen and an exine lacking spinules (Fig. 3).

## Discussion

Pollen grains of the representative species of the genera of tribe Hylocereeae studied here share the pollen type common to Caryophyllales: tricolpate to pantocolpate with the exine spinulose and perforate (Nowicke and Skvarla 1979). Furthermore, in particular for Cactaceae, tricolpate pollen has been described in all tribes of subfamilies Pereskioideae and Cactoideae (Lehuenberger 1976; Kurtz 1948, 1963) and is the most common pollen type in eudicots (Erdtman 1952; Furness and Rudall 2004).

From the 25 pollen characters analyzed, only seven attributes (five qualitative and two continuous) exhibited useful taxonomic variation. Four genera in Hylocereeae: *Epiphyllum*, *Acanthocereus*, *Disocactus*, *Selenicereus* (comprising *Hylocereus* and three species of *Weberocereus*), and *Aporocactus* have pollen grains with essentially similar morphology. That being said, *Disocactus* and *Epiphyllum* form part of the Phyllocactoid clade while *Selenicereus* and *Weberocereus* form part of the Hylocereoid clade in the molecular phylogeny constructed by Korotkova et al. (2017).

Despite the fact that the majority of taxa studied here share many pollen attributes, certain characters were common to limited groups of species. By way of example, *Kimnachia ramulosa* and *Pseudorhipsalis amazonica* are the only two species included in our study that share the attributes of brevicolpate pollen grains with small apertures. *Kimnachia* is a recently described genus whose sole species was previously included in *Pseudorhipsalis* (Korotkova et al. 2017). In addition, *Kimnachia ramulosa* and *Pseudorhipsalis amazonica* also share the character of pollen grains with 12–15 colpi with *Weberocereus.* Furthermore, *Selenicereus glaber* and *Weberocereus tunilla* share the character of convex quadrangular contour in polar view (Amb); *S. glaber* was previously included in *Weberocereus* (Barthlott and Hunt 1993).

Two species in *Selenicereus* (*S. minutiflorus* and *S. stenopterus)* stand out for lacking spinules in the exine in tribe Hylocereeae. They were retrieved in the *Hylocereus* clade in the plastid phylogeny of Korotkova et al. (2017) and transferred with all *Hylocereus* spp. to *Selenicereus*. They have remarkable morphology with miniature plants bearing pinkish flowers in contrast to the rest of the species in the current concept of *Selenicereus* whose flowers are white. Bauer (2003) transferred these two species from *Selenicereus* to *Hylocereus* and classified them in the *Salmdyckia* group of *Hylocereus*. Previously, Britton and Rose (1920) included these taxa (*S. minutiflorus* and *S. stenopterus*) in *Mediocactus*, a genus with intermediate morphological characteristics between *Selenicereus* and *Hylocereus*, with spines on the pericarpel.

The *Salmdyckia* group, including *S. ocamponis*, *S. setaceus* and *S. megalanthus*, possesses the largest pollen grains in the genus. Of these three species, *Selenicereus megalanthus* had the largest pollen grains, with a pollen grain size that could be correlated with polyploidy, a process that can produce large to very large pollen grains (Muller 1979). Chromosome counts for this species indicate that it is tetraploid (Tel-Zur et al. 2004; Tel-Zur et al. 2011).

Furthermore, multivariate analyses corroborated the results of discrete and qualitative characters, displaying species such as *Selenicereus megalanthus*, *S. stenopterus*, *S. multiflorus* and *Kimnachia ramulosa* as outliers in the multidimensional space. Variation in these analyses was found to be mainly associated with size (equatorial diameter, polar axis, and spinule dimensions).

Of the genera in Hylocereeae, *Selenicereus* in its current concept including the species previously considered in *Hylocereus* and three species formerly classified in *Weberocereus*, is the taxon with the greatest variation in pollen grains. For instance, it includes species with and without spinules in the exine, variable shape (subprolate to oblate-spheroidal), and polar area index is either small, medium or large. Moreover, the generic limits of *Hylocereus* and *Selenicereus* have changed over time (Gómez-Hinostrosa and Hernández 2014; Gómez-Hinostrosa et al. 2014; Cruz et al. 2016; Korotkova et al. 2017). Pollen size, the absence of spinules and the morphological characters in species such as *S. minutiflorus* and *S. stenopterus* suggest they might belong to a genus other than *Hylocereus* or *Selenicereus*.

Pollen research that concentrates on finding crucial taxonomical characters in Cactaceae has been scarce. Nevertheless, current studies in other plant groups have demonstrated their utility and that of other data sources (e.g. Kriebel et al. 2017; Niu et al. 2018; Siniscalchi et al. 2017; Wrońska-Pilarek et al. 2018). The most complete study on the palynology of the entire Cactaceae by Leuenberger (1976b) was published in a series of dissertations and is difficult to acquire. Probably the delicate pollen in Cactaceae which is difficult to process is one of the causes of the limited number of studies on pollen.

The pollen attributes identified here and that are shared by a number of species belonging to different genera that have recently been segregated or grouped together, suggests that additional evidence should be gathered and new phylogenetic analyses performed to clarify boundaries. Circumscription of the genera in tribe Hylocereeae has only been carried out based on a set of molecular or morphological characters. Our project on the *Hylocereus* clade will include the palynological characters determined here, along with other sources of attributes such as their morphological, ecological and molecular traits.

## Supplementary Material

XML Treatment for Acanthocereus

XML Treatment for Aporocactus

XML Treatment for Disocactus

XML Treatment for Epiphyllum

XML Treatment for Kimnachia

XML Treatment for Pseudorhipsalis

XML Treatment for Selenicereus

XML Treatment for Selenicereus (Hylocereus sect. Hylocereus

XML Treatment for Selenicereus

XML Treatment for Selenicereus

XML Treatment for Weberocereus

## Supplementary materials

Supplementary material 1 Table S1 Data type: species data Explanation note: Qualitative and quantitative morphological characters of pollen for the representative species in the tribe Hylocereeae. https://binary.pensoft.net/file/326052This dataset is made available under the Open Database License (http://opendatacommons.org/licenses/odbl/1.0/). The Open Database License (ODbL) is a license agreement intended to allow users to freely share, modify, and use this Dataset while maintaining this same freedom for others, provided that the original source and author(s) are credited.Catalina Ruiz-Domínguez, Andrew P. Vovides, Victoria Sosa
