# Supplementary material for: Systematic relevance of pollen morphology in tribe Hylocereeae (Cactaceae)
Source: PhytoKeys. 2019 Aug 19;128:121–40. doi: 10.3897/phytokeys.128.35842 (PMC6711936; doi:10.3897/phytokeys.128.35842)
Supplement: Supplementary material 1 [file phytokeys-128-121-s001.docx]

**Supplementary data**

**Table S1.** Median for eleven characters of pollen morphology of 27 species 8 genera of the tribe Hylocereeae. Equatorial diameter in polar view (EDP); Apocolpium (APO); Mesocolpium (MESO); Equatorial diameter in equatorial view (EDE); Polar axis (PA); Spinule length (SL); Spinule base (SB); Perforation diameter (PD) and Exine length (EL). PE ratio (Shape class) and PAI (Polar Area Index).

| **Species** | **EDP** | **APO** | **MESO** | **EDE** | **PA** | **SL** | **SB** | **PD** | **EL** | **PE** | **PAI** |
| --- | --- | --- | --- | --- | --- | --- | --- | --- | --- | --- | --- |
| *Acanthocereus chiapensis* | 79.19 | 24.95 | 63.24 | 52.29 | 67.76 | 1.43 | 1.35 | 0.24 | 3.76 | 1.30 | 0.32 |
| *Acanthocereus tetragonus* | 75.35 | 30.45 | 58.84 | 61.34 | 59.49 | 1.45 | 1.41 | 0.27 | 2.85 | 0.97 | 0.41 |
| *Aporocactus martianus* | 122.61 | 44.75 | 88.92 | 93.62 | 106.91 | 1.64 | 1.53 | 0.24 | 3.66 | 1.15 | 0.36 |
| *Disocactus ackermanii* | 124.07 | 41.84 | 89.61 | 89.57 | 105.36 | 1.75 | 1.47 | 0.51 | 3.64 | 1.19 | 0.34 |
| *Disocactus speciosus* | 146.83 | 50.61 | 102.95 | 105.46 | 119.01 | 1.51 | 1.44 | 0.72 | 2.91 | 1.13 | 0.35 |
| *Epiphyllum oxypetalum* | 104.91 | 33.35 | 82.68 | 81.63 | 86.82 | 1.82 | 1.21 | 0.54 | 2.42 | 1.07 | 0.32 |
| *Epiphyllum thomasianum* | 97.23 | 45.29 | 79.36 | 95.91 | 86.17 | 1.56 | 1.31 | 0.49 | 3.05 | 0.90 | 0.47 |
| *Kimnachia ramulosa* | 55.47 | 2.85 | 32.00 | 49.15 | 52.46 | 0.34 | 0.45 | 0.18 | 3.85 | 1.07 | 0.05 |
| *Pseudorhipsalis amazonica* | 78.13 | 49.16 | 51.69 | 81.71 | 81.24 | 0.79 | 0.48 | 0.12 | 3.20 | 0.99 | 0.63 |
| *Selenicereus alliodorus* | 105.35 | 43.10 | 79.51 | 73.64 | 80.94 | 1.49 | 1.54 | 0.28 | 2.91 | 1.12 | 0.41 |
| *Selenicereus costaricencis* | 104.72 | 39.29 | 78.38 | 73.16 | 86.12 | 1.36 | 1.13 | 0.44 | 2.53 | 1.18 | 0.38 |
| *Selenicereus escuintlensis* | 95.87 | 35.41 | 73.15 | 74.70 | 72.08 | 1.49 | 1.32 | 0.42 | 2.03 | 0.97 | 0.37 |
| *Selenicereus glaber* | 90.88 | 55.11 | 58.64 | 87.13 | 90.81 | 1.42 | 1.63 | 0.39 | 3.30 | 1.05 | 0.61 |
| *Selenicereus grandiflorus* | 118.10 | 43.87 | 91.21 | 97.88 | 104.35 | 1.28 | 1.14 | 0.26 | 2.68 | 1.07 | 0.37 |
| *Selenicereus guatemalensis* | 82.47 | 25.13 | 66.11 | 69.98 | 72.39 | 1.33 | 1.18 | 0.49 | 2.25 | 1.04 | 0.31 |
| *Selenicereus hamatus* | 98.40 | 35.85 | 73.86 | 72.73 | 76.65 | 1.86 | 1.38 | 0.37 | 2.76 | 1.06 | 0.37 |
| *Selenicereus megalanthus* | 154.42 | 61.98 | 93.95 | 114.35 | 115.53 | 1.78 | 1.24 | 0.52 | 3.65 | 1.01 | 0.40 |
| *Selenicereus minutiflorus* | 62.78 | 37.41 | 49.85 | 60.22 | 52.18 | 0.00 | 0.00 | 0.63 | 3.33 | 0.87 | 0.60 |
| *Selenicereus monacanthus* | 90.10 | 30.70 | 71.00 | 74.26 | 81.90 | 1.48 | 1.16 | 0.42 | 2.58 | 1.11 | 0.34 |
| *Selenicereus ocamponis* | 130.74 | 39.88 | 97.74 | 89.08 | 103.15 | 1.41 | 1.02 | 0.37 | 2.40 | 1.17 | 0.31 |
| *Selenicereus polyrhizus* | 81.02 | 27.47 | 62.68 | 64.09 | 68.38 | 1.51 | 1.27 | 0.39 | 2.95 | 1.07 | 0.34 |
| *Selenicereus setaceus* | 137.56 | 46.15 | 104.50 | 108.28 | 118.31 | 1.54 | 1.19 | 0.42 | 2.49 | 1.10 | 0.34 |
| *Selenicereus sp.* | 102.75 | 35.65 | 78.27 | 85.97 | 95.21 | 1.69 | 1.08 | 0.45 | 3.42 | 1.11 | 0.35 |
| *Selenicereus stenopterus* | 102.35 | 39.20 | 75.03 | 76.56 | 88.96 | 0.00 | 0.00 | 0.41 | 2.91 | 1.17 | 0.38 |
| *Selenicereus triangularis* | 114.54 | 51.10 | 87.45 | 81.94 | 74.22 | 1.34 | 1.00 | 0.29 | 3.08 | 0.91 | 0.45 |
| *Selenicereus undatus* | 101.15 | 39.81 | 78.02 | 73.72 | 76.61 | 1.66 | 0.93 | 0.36 | 2.60 | 1.04 | 0.40 |
| *Weberocereus tunilla* | 93.93 | 52.79 | 56.49 | 82.95 | 88.74 | 1.41 | 1.66 | 0.28 | 2.44 | 1.07 | 0.57 |
